# Reduction of oxidative stress suppresses poly-GR-mediated toxicity in zebrafish embryos

**DOI:** 10.1242/dmm.049092

**Published:** 2021-12-01

**Authors:** Fréderike W. Riemslagh, Rob F. M. Verhagen, Esmay C. van der Toorn, Daphne J. Smits, Wim H. Quint, Herma C. van der Linde, Tjakko J. van Ham, Rob Willemsen

**Affiliations:** Department of Clinical Genetics, Erasmus University Medical Center Rotterdam, 3000 CA, Rotterdam, The Netherlands

**Keywords:** *C9ORF72*, Amyotrophic lateral sclerosis, Frontotemporal dementia, Poly-GR, Neurodegeneration, Oxidative stress

## Abstract

The hexanucleotide (G_4_C_2_)-repeat expansion in the *C9ORF72* gene is the most common pathogenic cause of frontotemporal dementia (FTD) and amyotrophic lateral sclerosis (ALS). This repeat expansion can be translated into dipeptide repeat proteins (DPRs), and distribution of the poly-GR DPR correlates with neurodegeneration in postmortem C9FTD/ALS brains. Here, we assessed poly-GR toxicity in zebrafish embryos, using an annexin A5-based fluorescent transgenic line (secA5) that allows for detection and quantification of apoptosis *in vivo*. Microinjection of RNA encoding poly-GR into fertilized oocytes evoked apoptosis in the brain and abnormal motor neuron morphology in the trunk of 1-4-days postfertilization embryos. Poly-GR can be specifically detected in protein homogenates from injected zebrafish and in the frontal cortexes of C9FTD/ALS cases. Poly-GR expression further elevated MitoSOX levels in zebrafish embryos, indicating oxidative stress. Inhibition of reactive oxygen species using Trolox showed full suppression of poly-GR toxicity. Our study indicates that poly-GR can exert its toxicity via oxidative stress. This zebrafish model can be used to find suppressors of poly-GR toxicity and identify its molecular targets underlying neurodegeneration observed in C9FTD/ALS.

## Abstract (precis)

**Summary:** Toxicity of C9ALS/FTD poly-GR in zebrafish embryos is suppressed by Trolox, and poly-GR can be detected and quantified in zebrafish model protein homogenates, and in the frontal cortex of C9FTD/ALS cases.

## INTRODUCTION

Frontotemporal dementia (FTD) and amyotrophic lateral sclerosis (ALS) are two neurological disorders that are characterized by the degeneration of cortical neurons in the frontal and temporal cortices and motor neurons in the motor cortex and spinal cord (Burrell et al., 2016). The hexanucleotide (G_4_C_2_)-repeat expansion in the *C9ORF72* gene is a shared genetic factor that has been linked to both FTD and ALS (DeJesus-Hernandez et al., 2011; Renton et al., 2011). Patients carrying repeat expansions can develop symptoms of both disorders (Burrell et al., 2016), and this combination of symptoms/disorders has been termed C9FTD/ALS. The G_4_C_2_-repeat expansion can cause neurodegeneration via both loss- and gain-of-function mechanisms, or a combination of both (Balendra and Isaacs, 2018). Methylation of the repeat and surrounding CpG islands can silence the *C9ORF72* gene, leading to haploinsufficiency (Balendra and Isaacs, 2018). Sense and antisense RNA containing the expanded repeat accumulate in RNA foci and sequester multiple RNA-binding proteins (Balendra and Isaacs, 2018). Furthermore, dipeptide repeat proteins (DPRs) are produced by unconventional repeat-associated non-AUG (RAN) translation of the repeat sequences in both sense and antisense directions, creating different DPRs: poly-glycine-alanine (GA), poly-glycine-proline (GP), poly-glycine-arginine (GR), poly-proline-arginine (PR), poly-proline-alanine (PA) and poly-proline-glycine (PG) (Mori et al., 2013; Ash et al., 2013; Zu et al., 2013; Gendron et al., 2013). These DPRs are found throughout postmortem brain tissue of patients (Mackenzie et al., 2013) but only poly-GR pathology correlates with neurodegeneration (Saberi et al., 2018; Sakae et al., 2018; Quaegebeur et al., 2020).

Notably, the arginine-containing DPRs poly-GR and poly-PR have been reported to be very toxic in both cell and animal models (Mizielinska et al., 2014; Wen et al., 2014; Swaminathan et al., 2018; Tao et al., 2015; Kanekura et al., 2016; Lee et al., 2016; Zhang et al., 2018a). Poly-GR and poly-PR interact with ribosomal proteins, heterochromatin, nucleolar proteins, RNA-binding proteins and proteins containing low-complexity domains (LCDs) (Lee et al., 2016; Lin et al., 2016; Tao et al., 2015; Kanekura et al., 2016; Zhang et al., 2018a, 2019). LCD proteins can form membraneless organelles, such as nucleoli, the nuclear pore and stress granules. Poly-GR and poly-PR have been shown to alter the dynamics and assembly of these organelles, leading to reduced mRNA translation, ribosomal stress, endoplasmic reticulum (ER) stress and aberrant nucleocytoplasmic transport (Boeynaems et al., 2017; Zhang et al., 2018a,b; Lee et al., 2016; Freibaum et al., 2015). In addition, overexpression of either poly-GR or poly-PR resulted in reduced translation in NSC34 and HeLa cells (Kanekura et al., 2016; Rossi et al., 2015), and in a mouse model for poly-GR (Zhang et al., 2018a). Furthermore, poly-GR can interact with mitochondrial ribosomal proteins and consequently impair mitochondrial function in C9FTD/ALS induced pluripotent stem cell (iPSC)-derived neurons and in a recent mouse model (Lopez-Gonzalez et al., 2016; Dafinca et al., 2016; Choi et al., 2019). Finally, poly-GR increases the amount of oxidative stress, which can cause DNA damage in motor neurons differentiated from iPSCs (Lopez-Gonzalez et al., 2016). Importantly, as all these processes can influence each other and lead to general cellular malfunctioning, the primary starting point of the toxicity cascade is still unknown and warrants further investigation (Zhang et al., 2018b; Kwon et al., 2014).

Multiple cell and animal models, including zebrafish (Fortier et al., 2020), have been generated for C9FTD/ALS, many of which support DPR toxicity and a gain-of-function hypothesis (reviewed by Balendra and Isaacs, 2018; Batra and Lee, 2017; Gendron and Petrucelli, 2018; Fortier et al., 2020). New therapeutical strategies are under development, which depend on reliable *in vivo* drug screens (Jiang and Ravits, 2019). However, not all *C9ORF72* animal models show a robust phenotype, including neurodegeneration, which is needed for quantifiable pharmaceutical outcome measures. Here, we generated a zebrafish (*Danio rerio*) vertebrate animal model to quantify and screen suppressors of poly-GR toxicity *in vivo*. Zebrafish have been extensively used in (neuro)toxicity studies for multiple reasons (Van Houcke et al., 2015; Wiley et al., 2017). Zebrafish embryos are transparent and develop externally, allowing for easy detection of body and organ abnormalities. Basic (neuro)biological processes are conserved between vertebrates, making identification of aberrant cellular and molecular pathways in the pathogenesis of human disorders feasible (Van Houcke et al., 2015; Santoriello and Zon, 2012). Finally, zebrafish are well suited for drug screens because they readily take up small molecules from the water (Wiley et al., 2017).

To address poly-GR toxicity in zebrafish embryos, we injected RNA encoding ATG-mediated codon-optimized 100×GR. High concentrations led to dead and malformed fish, whereas low concentrations of only 10 pg poly-GR were sufficient to evoke apoptosis in the brain and caused aberrant motor neuron morphology at 1-4 days postfertilization (dpf). To visualize and quantify apoptosis, we made use of transgenic zebrafish expressing fluorescently labeled SecA5, which labels apoptotic clusters with YFP (van Ham et al., 2010). This transgenic model allows screening for drugs that modify poly-GR toxicity, and as a proof of concept we identify Trolox as a suppressor of GR-toxicity. Trolox is an antioxidant that reduces reactive oxygen species (ROS), which are free radicals that can evoke oxidative stress and cause mitochondrial and DNA damage, both of which are linked to many neurodegenerative diseases (Bhat et al., 2015). In this study, we highlight the involvement of this cellular stress pathway in the pathogenesis of *C9ORF72*-linked ALS and FTD.

## RESULTS

### Injection of poly-GR-encoding RNA evokes apoptosis and aberrant motor neuron axon morphology in 1-4-dpf zebrafish embryos

**Fig. 1.**
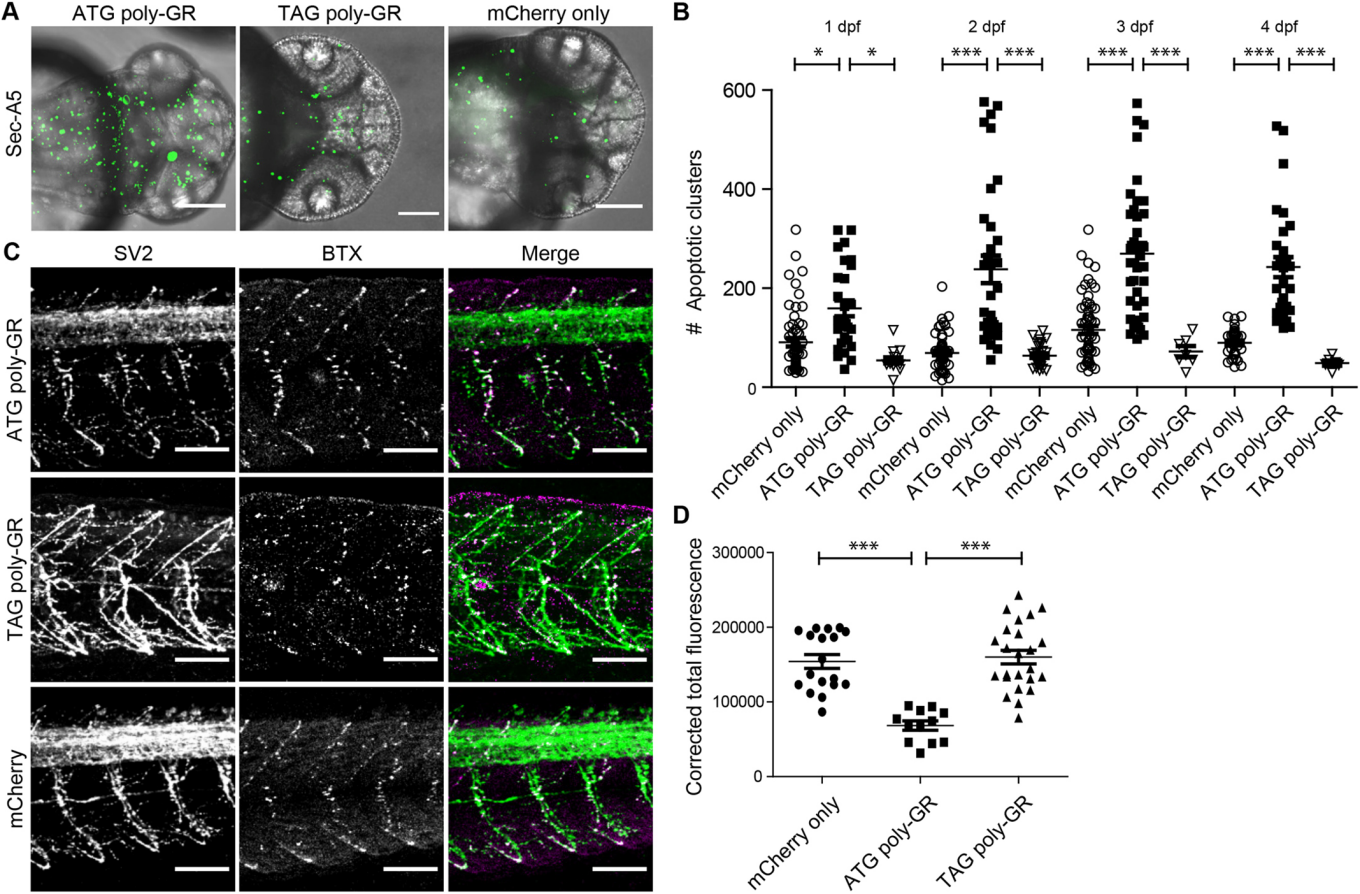
**Injection of ATG poly-GR evokes apoptosis and aberrant motor neuron axon morphology in 1-4-dpf zebrafish embryos.** (A) Maximum projection of *z*-stack images of the SecA5 fluorescent reporter line (green) embryos 48 h after injection with 10 pg ATG poly-GR, 10 pg TAG poly-GR or 400 pg mCherry only. (B) Quantification of *z*-stack images of the SecA5 fluorescent reporter line embryos after injection with 10 pg ATG poly-GR, 10 pg TAG poly-GR or 400 pg mCherry only at 1-4 dpf. *N*=minimum of ten fish per group (mean±s.e.m.). *P*<0.0001 (Kruskal–Wallis test). *P<0.05, ***P<0.0001 (Dunn's post-hoc multiple comparison test), differences of ATG poly-GR-injected fish compared to 10 pg TAG poly-GR and mCherry only at 1-4 dpf. (C) Synaptic vesicle (SV2) in combination with α-bungarotoxin (BTX) staining demonstrates aberrant axonal protrusions in the tail of 2-dpf wild-type AB embryos after injection with 10 pg ATG poly-GR compared to 10 pg TAG poly-GR and 400 pg mCherry only. *n*=10 per group (mean±s.e.m.). (D) Fluorescence of SV2 staining was measured for five fish/group and three neurites per fish, and was significantly reduced in 10 pg ATG poly-GR-injected embryos. *P*<0.0001 (one-tailed *t*-test with Welch's correction). Scale bars: 100 µm.

We began by assessing the toxicity of poly-GR at the systemic level. RNA was transcribed *in vitro* from an ATG-100×GR construct, using alternative codons without the G_4_C_2_-repeat sequence. This allows for testing poly-GR DPR toxicity independent of possible RNA toxicity caused by pure G_4_C_2_ repeats. A concentration series ranging from 1 to 200 pg of RNA was injected into the yolk sac of fertilized zebrafish oocytes at the one-cell stage and showed a dose-dependent toxicity (Fig. S1), with high concentrations leading to dead and undeveloped embryos and lower concentrations leading to malformations, including heart edema and bent tails. For subsequent experiments, we chose a dose of 10 pg poly-GR encoding RNA that resulted in a robust and reproducible phenotype without causing severe malformations. The 10-pg dose of poly-GR injections still resulted in 30% of injected embryos with visible deformity (including heart edema and bent tails), but only those with lack of visible deformity were used. Lower concentrations did not result in a clear and reproducible phenotype in terms of apoptotic cluster count (Fig. S1). To determine the effect of poly-GR at the cellular level, we used the SecA5 reporter zebrafish line that fluorescently labels apoptotic clusters *in vivo* with YFP (van Ham et al., 2010). Cells labeled with secA5-YFP are apoptotic and exhibit several hallmarks of apoptotic cells, including DNA fragmentation, nuclear condensation, altered morphology and loss of membrane asymmetry (van Ham et al., 2010). As injections by themselves already cause a slight increase of cell death, we used RNA from an mCherry-only construct as control. This marker was also used to select for correctly injected fish. Co-injection of mCherry RNA together with low concentrations of poly-GR encoding RNA evoked a 1.5-2.5-fold increase in apoptosis in zebrafish embryos at all stages (1-4 dpf) (Fig. 1A). The increase in apoptosis was confirmed by TUNEL staining (Fig. S2). To quantify the number of apoptotic clusters, we imaged zebrafish heads *in vivo* and automatically quantified the YFP^+^ clusters in *z*-stack images. A dose of 10 pg of poly-GR RNA was sufficient to cause a significant increase in the number of apoptotic clusters in the brain of zebrafish embryos at 1-4 dpf compared to mCherry control-injected fish (Fig. 1B, Kruskal–Wallis test Dunn's post-hoc multiple comparison test, *P*<0.0001), without generating gross morphological abnormalities or affecting viability. TUNEL quantification also showed a significant difference between poly-GR-injected fish versus mCherry only (Fig. S2). To further characterize the effect of poly-GR overexpression, we stained embryos for synaptic vesicle 2 (SV2) in combination with α-bungarotoxin (α-BTX), to visualize axons and the neuromuscular junction. This revealed that poly-GR-injected zebrafish embryos display aberrant neuromuscular morphology with fewer axonal protrusions of the motor neuron axonal structure in the trunk (Fig. 1C). SV2 intensity was significantly reduced in poly-GR-injected embryos (Fig. 1D, one-tailed *t*-test with Welch's correction, *P*<0.0001).

### Injected poly-GR RNA is translated into poly-GR peptide, which remains detectable for at least 4 days *in vivo*

**Fig. 2.**
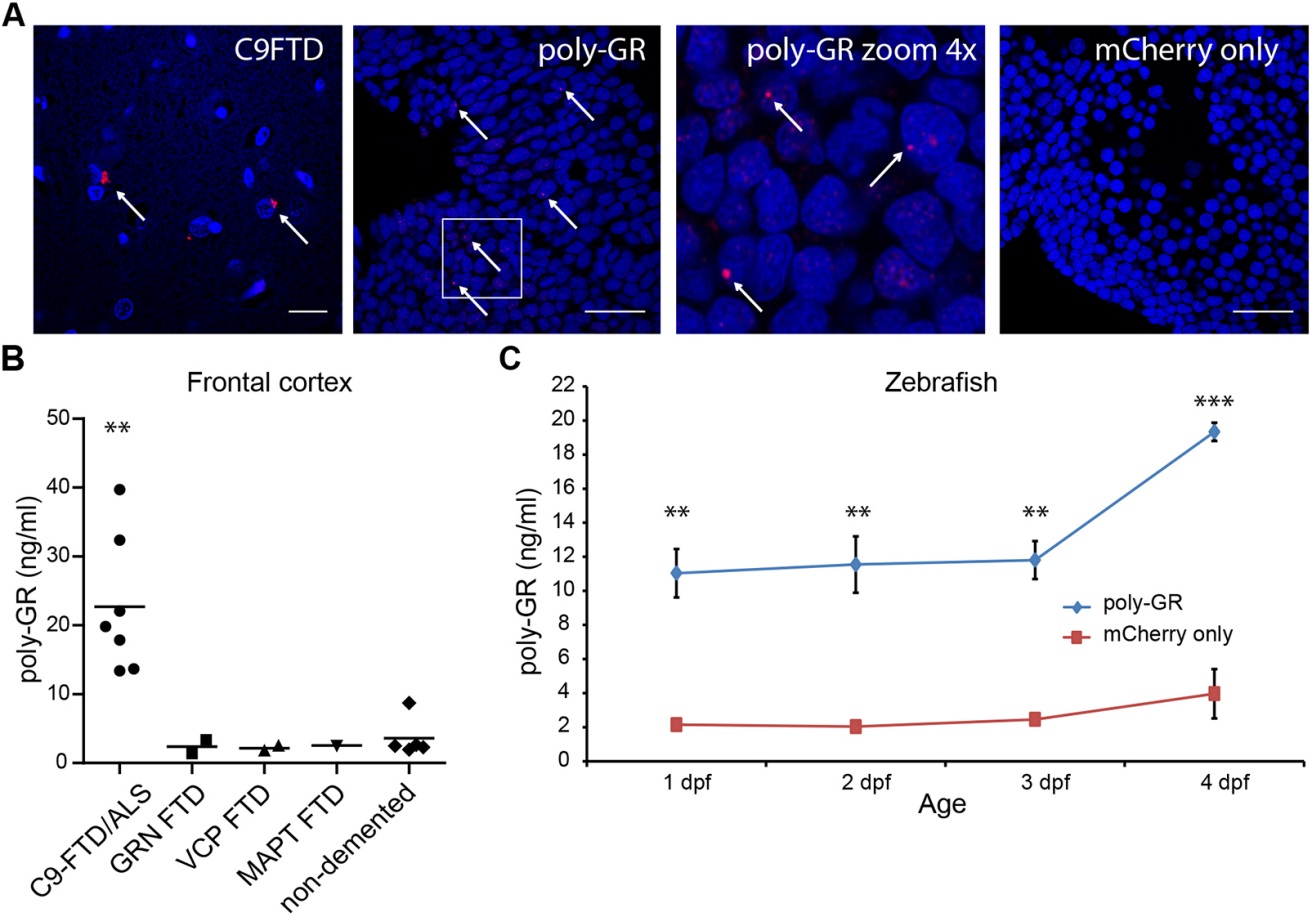
**Poly-GR peptides are detected as (peri)nuclear puncta in zebrafish embryos at 1-4 dpf.** (A) Immunofluorescence staining for poly-GR (red) in 2 dpf old wild-type AB embryos after injection with 10 pg poly-GR or 400 pg mCherry only. *N*=30 per group. C9FTD patient frontal cortex sections were used as a positive control. Poly-GR peptides were detected as nuclear or perinuclear dots in all poly-GR-injected fish (arrows). Nuclei were stained with Hoechst. Scale bars: 20 µm. (B) Poly-GR is detected in postmortem frozen brain samples of C9ORF72 FTD/ALS cases (*n*=7) but not in postmortem frozen brain samples of FTD patients with mutations in progranulin (GRN; *n*=2), Valosin-containing protein (VCP; *n*=2), microtubule-associated protein tau (MAPT, *n*=1) or in brain samples of non-demented controls (*n*=5) (mean±s.e.m.). One-way ANOVA (*P*=0.0024) with post Tukey's test indicating a difference between the C9ORF72 FTD/ALS group and all other groups. (C) ELISA for the detection of poly-GR shows a signal of 10-20 pg peptide in 1-4 dpf wild-type AB embryos injected with 10 pg RNA encoding poly-GR. Two-way ANOVA (*P*=0.0003) with post Bonferroni test indicating that all timepoints were significantly different from mCherry-only-injected fish. *n*=90 fish per group per timepoint divided over three independent experiments (30 fish per group×three experiments per timepoint). *P<0.05, ***P<0.0001.

To confirm the translation of injected RNA encoding poly-GR, peptides were visualized using immunofluorescent staining of whole-body zebrafish at 1-4 dpf. Poly-GR peptides were detected as nuclear and perinuclear puncta in postmortem frontal cortex of *C9ORF72* FTD cases, and in embryos injected with 10 pg poly-GR, but were absent in mCherry-only-injected embryos and in human frontal cortex from non-demented controls (Fig. 2A; Fig. S3A). To quantify poly-GR peptide expression, we developed an ELISA using a synthetic 15×GR peptide (LifeTein). This ELISA showed high specificity for poly-GR and did not show any signal for a 15×PR synthetic peptide (LifeTein) (Fig. S3B). Next, we diluted the 15×GR peptide to make a dose-response curve in the high and low range (Fig. S3C), which revealed a sensitivity of 200 pg/ml (Fig. S3C). To further validate the ELISA, we isolated protein from the frontal cortex of seven C9FTD/ALS cases, five FTD cases resulting from other genetic causes (*GRN*, *VCP* or *MAPT*) and five non-demented controls (Fig. 2B). Poly-GR could only be detected in frontal cortex samples of C9FTD/ALS cases, illustrating the specificity of our assay [one-way ANOVA (*P*=0.0024) with a post Tukey's test indicating a difference between the C9FTD/ALS group and all other groups]. The calculated amount of poly-GR was, on average, 22.7 ng/ml in the frontal cortices of C9FTD/ALS patients. Additional protein isolation using 20% SDS and 95°C incubation to dissolve more poly-GR from the insoluble fraction only yielded 1.28 ng/ml extra poly-GR (Fig. S3). Zebrafish embryos injected with 10 pg of RNA encoding poly-GR showed a signal of 10-20 pg/ml in the ELISA that was significantly different from Cherry-only-injected fish (two-way ANOVA, *P*=0.0003) and remained high for 1-4 days after injection (Fig. 2C), whereas poly-GR mRNA was mostly gone after 2 dpf (Fig. S3F). Together, the ELISA data confirmed that our poly-GR zebrafish model mimics physiological poly-GR levels observed in C9FTD/ALS patient material.

To further study the specificity of the observed protein toxicity, we generated a translation-defective 100×GR construct by mutating the ATG start codon into a TAG stop codon. This construct prevents the generation of poly-GR production and can be considered as a control for RNA toxicity per se. We confirmed that this TAG-100×GR did not produce any poly-GR peptides using our ELISA for poly-GR (Fig. S3D). Injection of high amounts (50-200 pg) of RNA of the TAG-100×GR construct caused an increase in dead and malformed embryos (Fig. S1). Apparently, a high concentration of non-coding RNA can have a small toxic effect on its own, even though this RNA does not contain a G_4_C_2_-repeat sequence. Low amounts (2-10 pg) of TAG-mediated poly-GR RNA was only slightly toxic compared to mCherry only (Fig. S1), whereas low amounts of ATG-mediated poly-GR RNA evoked abundant apoptosis (Fig. 1). In summary, injection of poly-GR RNA is translated into poly-GR peptides that present as (peri)nuclear puncta throughout the zebrafish body and remain detectable for at least 4 days. Expression of poly-GR peptides causes abundant apoptosis in the developing zebrafish.

### Inhibition of oxidative stress rescues poly-GR-mediated toxicity *in vivo*

**Fig. 3.**
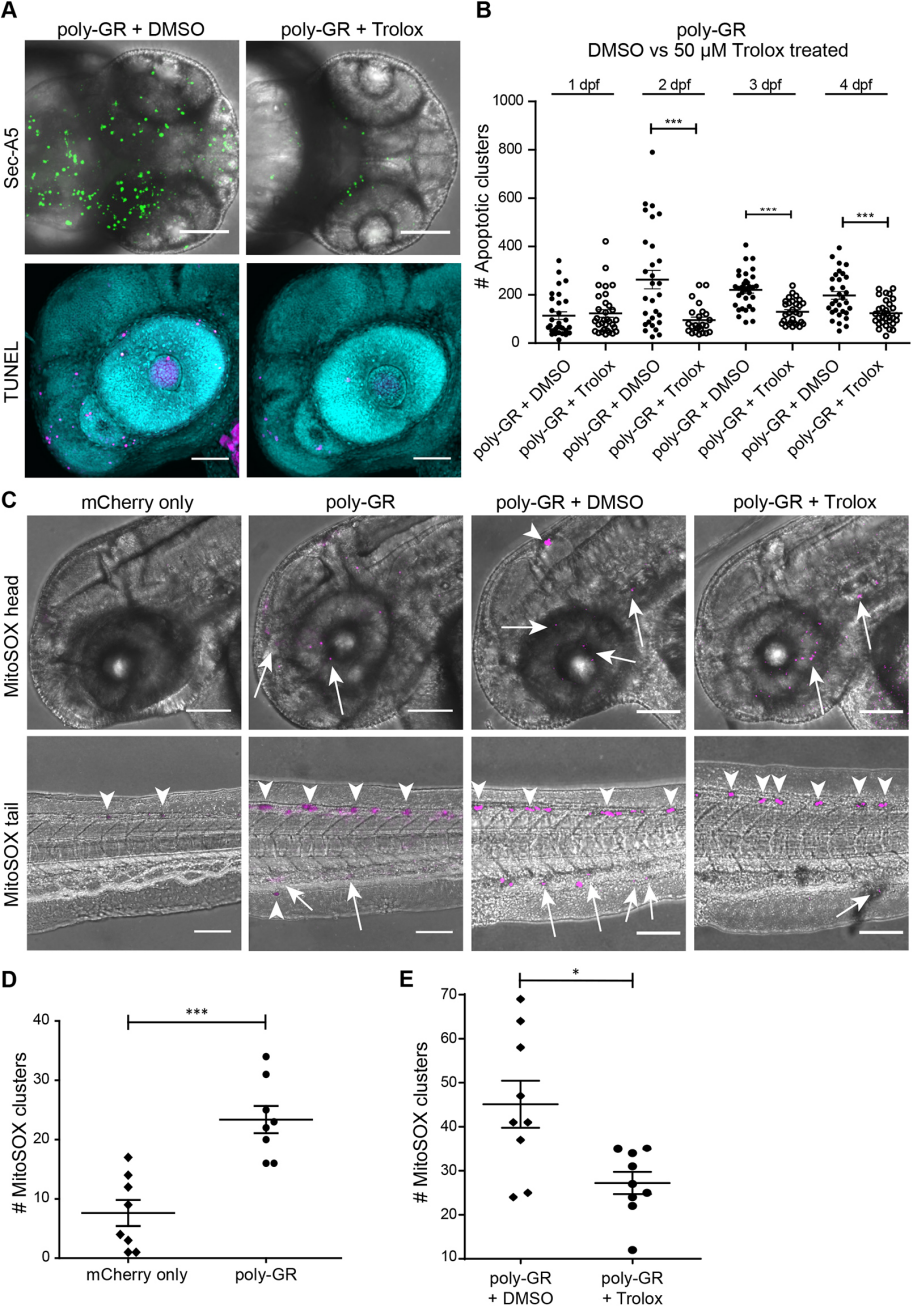
**Trolox reduces the number of apoptotic clusters and oxidative stress in zebrafish embryos injected with 10 pg poly-GR.** (A) Upper panel: maximum projection of *z*-stack images of the SecA5 fluorescent reporter line embryos 48 h after injection with 10 pg poly-GR and treated with 50 µM Trolox or DMSO only. Lower panel: maximum projection of TUNEL staining of the same treatment groups in wild-type AB fish at 2 dpf. (B) Quantification of SecA5 *z*-stack images (mean±s.e.m.). *N*=30 fish per group per day. Kruskal–Wallis test (*P*<0.0001). Dunn's post-hoc multiple comparison test (***P<0.0003). (C) MitoSOX red staining in 2 dpf wild-type AB embryos after injection with 10 pg poly-GR or 400 pg mCherry only, and treated with DMSO only or Trolox (dissolved in DMSO). MitoSOX signal (magenta) is seen as clusters in the brain and in the spinal cord in the tail. MitoSOX staining also shows ROS reactivity in pigmented cells (arrowheads) and along the tail and head (arrows). (D) MitoSOX signal is significantly higher in 10 pg poly-GR compared to mCherry-only-injected zebrafish embryo tails at 2 dpf. *N*=8 fish per group (mean±s.e.m.). Two-tailed unpaired Student's *t*-test (*P*=0.0002). *F*-test for equal variances (*P*=0.914) demonstrated that variances were not significantly different. (E) MitoSOX signal is significantly reduced in 10 pg poly-GR-injected zebrafish embryo tails treated with Trolox versus DMSO only at 2 dpf. *N*=9 fish per group (mean±s.e.m.). *F*-test (*P*=0.045) demonstrated that variances were significantly different. Two-tailed unpaired Student's *t*-test with Welch's correction *P*=0.01. H_2_O_2_-treated embryos used for MitoSOX positive control experiments, with an additional *n*=8 fish per group, are shown in Fig. S4. **P<0.001, ***P<0.0001. Scale bars: 100 µm.

Poly-GR peptides have been shown to disturb many cellular processes and pathways (Balendra and Isaacs, 2018; Jiang and Ravits, 2019; Gendron and Petrucelli, 2018) but their primary target is still unknown. To discriminate between primary and secondary effects, we used a pharmacological approach. In *C9ORF72* patient iPSC-derived motor neurons that express both poly-GR and poly-PR, decreased cell survival is correlated with dysfunction in Ca^2+^ homeostasis, increased ER stress and reduced mitochondrial membrane potential (Dafinca et al., 2016). Trolox, an antioxidant that reduces ROS, partially rescued toxicity in an iPSC-induced motor neuron model of C9FTD/ALS (Lopez-Gonzalez et al., 2016). In our *in vivo* study, Trolox significantly reduced poly-GR-mediated apoptosis in Sec-A5 transgenic embryos (Fig. 3A,B) [Kruskal–Wallis test, *P*<0.0001. Dunn's post-hoc multiple comparison test (*P*<0.0001, difference between DMSO- and Trolox-treated fish at 2-4 dpf)]. Quantification of TUNEL staining also showed a reduction of apoptotic cells in zebrafish embryos treated with Trolox (Fig. 3A lower panel; Fig. S2; one-way ANOVA, *P*<0.0001. Post Tukey's test showed a significant difference between poly-GR-injected fish treated with Trolox compared to DMSO). As Trolox is known to inhibit the formation of ROS, we used a wholemount protocol to quantify the ROS in living zebrafish by MitoSOX staining. As pigmented cells are highly reactive to this assay, hydrogen peroxide was used as positive control to distinguish specific signal (Fig. S4). Indeed, 10 pg poly-GR-injected fish showed an increase in MitoSOX staining compared to mCherry-only-injected fish (Fig. 3C,D; Fig. S4). Trolox is dissolved in DMSO, so we treated poly-GR-injected fish with Trolox or an equal amount of DMSO and found that DMSO increased the amount of ROS even further, which was subsequently reduced by Trolox (Fig. 3C,E). Surprisingly, SecA5 and TUNEL apoptotic cell counts in Trolox-treated embryos were reduced to the baseline level of mCherry-only injections, consistent with the complete suppression of poly-GR toxicity by Trolox.

## DISCUSSION

In this study, we use zebrafish embryos to visualize and quantify apoptosis in brain tissue evoked by the injection of RNA encoding poly-GR. Poly-GR peptides were detectable as small (peri)nuclear puncta throughout the zebrafish body and their expression level slowly increased from 10 ng/ml to 20 ng/ml over 4 days, as quantified by ELISA, which mimics physiological levels seen in C9FTD/ALS patients. Poly-GR evoked the formation of ROS, which we were able to suppress using Trolox, an inhibitor of oxidative stress. Our study indicates the importance of this specific cellular stress pathway in the toxicity of poly-GR *in vivo*, and potentially also in the pathogenesis of C9FTD/ALS.

Our model only focuses on toxicity induced by a single DPR and does not take into account the effects of G_4_C_2_-repeat RNA toxicity, haploinsufficiency of the normal C9ORF72 protein or simultaneous expression of different DPRs. The construct is ATG mediated and uses alternative codons to encode DPRs to circumvent G_4_C_2_ RNA toxicity, and as a control we used an ATG-mutated translation-deficient construct to control for toxicity mediated by RNA alone. The injection of high RNA concentrations of this TAG construct was toxic in embryos, showing that high expression of RNA molecules per se has a toxic effect. The TAG construct used in our study does not give any information on the RNA toxicity of the pure G_4_C_2_-repeat structure. Thus, our model only provides information about poly-GR toxicity, which was clearly higher than the sole effect of injections of the TAG-construct.

In this study, we show that poly-GR can be specifically detected in brain sections and protein isolates from frontal cortices of C9FTD/ALS cases using a monoclonal antibody against poly-GR. This antibody is able to recognize both the cytoplasmic form of poly-GR and (peri)nuclear poly-GR inclusions in postmortem brain tissue (Fig. S3). After injections, poly-GR mRNA is present for 2 days and thereafter decreases (as expected for transient injections), but the amount of poly-GR protein slowly increases until 4 dpf. We think that poly-GR peptides that have formed in the previous days are more stable over time (the half-life of the poly-GR protein is longer than that of the mRNA). Possible aggregation of poly-GR might also affect the ELISA readout. In our injected zebrafish, poly-GR was mainly detected as nuclear puncta in the brain, whereas the observed poly-GR pathology in postmortem brain tissue of C9FTD/ALS patients mainly consists of cytoplasmic and perinuclear aggregation. Interestingly, solubility of DPRs can differ per brain region, and soluble DPRs are less abundant in clinically affected areas (Quaegebeur et al., 2020). Arginine methylation of poly-GR may also influence its cellular targets and might affect disease course (Gittings et al., 2020). Furthermore, poly-GR aggregation can be influenced by co-expression of poly-GA (Yang et al., 2015), which is not expressed in our model. For poly-GA, toxicity was directly linked to its cytoplasmic aggregation in a mouse model and neuronal cell culture (Zhang et al., 2016). For poly-GR, cytoplasmic aggregation does not seem to be necessary to exert its toxic effect in our model and in published mouse models (Zhang et al., 2018a; Choi et al., 2019), making this antibody and zebrafish model applicable for studying the toxicity of both aggregated and soluble poly-GR forms.

In the adult brain, apoptosis is linked to neurodegeneration (Yuan and Yankner, 2000), and signs of apoptosis have been shown in spinal cord motor neurons of ALS patients (Yoshiyama et al., 1994; Pedersen et al., 2000) and in neurons and astrocytes in brain tissue from FTD patients (Su et al., 2000). We observed increased apoptosis in zebrafish brain tissue upon expression of poly-GR. The SecA5 quantification in our model was higher than the TUNEL quantification, possibly because SecA5 labels the phospholipid phosphatidylserine layer that is exposed early during apoptosis. In the final stages of apoptosis, during which DNA fragmentation is greatest, apoptotic cells lack a defined cell membrane and nuclear architecture, and debris of multiple secA5-YFP^+^ cells can be observed near high-intensity TUNEL staining, making it possible to count more early apoptotic YPF^+^ cells than late apoptotic TUNEL^+^ cells (van Ham et al., 2010). Both quantifications showed a clear increase in poly-GR-injected zebrafish, confirming the toxicity of this peptide. Next to apoptosis in brain tissue, overexpression of DPRs in zebrafish models has been reported to cause motor axon outgrowth defects, such as shorter axons and aberrant branching (Swaminathan et al., 2018; Swinnen et al., 2018; Shaw et al., 2018). Our study confirms these findings and shows similarities to motor axonal phenotypes in zebrafish models for other genetic causes of FTD and ALS (Laird et al., 2010; Sakowski et al., 2012). Interestingly, overexpression of poly-GA did evoke toxicity but no defects in motor neurons or motility in zebrafish (Ohki et al., 2017; Swinnen et al., 2018), indicating that this phenotype cannot be generalized to all DPRs.

Our results suggest that inhibition of oxidative stress can suppress poly-GR toxicity. We were surprised to find a full rescue of poly-GR toxicity, indicating a primary or central role of oxidative stress in the pathogenesis evoked by poly-GR. Poly-GR can probably evoke oxidative stress via multiple ways. A recent poly-GR mouse model confirmed direct poly-GR binding to ATP5A1, a subunit of mitochondrial respiratory chain complex V (Choi et al., 2019), and poly-GR can act as a mitochondria-targeting signal (Li et al., 2020a). In *Drosophila*, poly-GR impairs mitochondrial inner membrane structure, ion homeostasis, mitochondrial metabolism and muscle integrity (Li et al., 2020b). Mitochondrial and/or oxidative stress can worsen ER stress by reducing the efficiency of protein folding pathways and thereby increasing the amount of misfolded proteins (Chong et al., 2017). Conversely, ER stress can cause ROS production (Zeeshan et al., 2016) and disrupt the membrane of mitochondria, as the two organelles are interconnected (Lau et al., 2018). Recent reports on mouse models for poly-GR and poly-PR show a downregulation of genes involved in ribosome biogenesis (Zhang et al., 2019), and poly-GR and poly-PR inhibit translation in cell culture (Zhang et al., 2018a; Kanekura et al., 2016; Moens et al., 2019; Radwan et al., 2020). Enhanced phosphorylation of eIF2α increased levels of ER foldase PDIA1, and upregulation of CHOP has been found in postmortem brain sections of ALS patients (Atkin et al., 2008; Ilieva et al., 2007; Ito et al., 2009; Matus et al., 2013), all indicating ER stress. Finally, our observed effect of Trolox mimics that of edaravone, a US Food and Drug Administration-approved drug for ALS that is believed to act as a ROS scavenger and decrease the generation of ROS (Yoshino and Kimura, 2006). The effectivity of these drugs and the widespread ER stress indicators found in multiple ALS models and patient material all point towards the involvement of these pathways in the pathogenesis of C9FTD/ALS.

The ability of poly-GR to induce abundant apoptosis in the absence of the other DPRs, G_4_C_2_-repeat RNA toxicity and haploinsufficiency illustrates the high toxicity of this specific DPR. To date, the effect of Trolox on the toxicity of DPRs has only been investigated in cell culture (Lopez-Gonzalez et al., 2016; Zhang et al., 2018b; Kramer et al., 2018). Our results suggest Trolox can also rescue DPR toxicity *in vivo*. Previous studies indicate that poly-GR might share cellular targets with poly-PR, including mitochondrial and ribosomal proteins (Lopez-Gonzalez et al., 2016; Dafinca et al., 2016; Moens et al., 2019; Kanekura et al., 2016). Therefore, it would be interesting to test whether Trolox can suppress both DPRs. Further screening experiments in poly-GR-expressing zebrafish embryos could yield additional small molecule suppressors of poly-GR toxicity. Our study shows that the reduction of oxidative stress can suppress poly-GR toxicity in zebrafish embryos and indicates a possible role for oxidative stress in the neurodegeneration observed in C9FTD/ALS patients.

## MATERIALS AND METHODS

### Sample collection

FTD/ALS and non-demented control human brain sections were provided by the Dutch Brain Bank. Patients or relatives provided informed consent for autopsy and the use of brain tissue for research purposes. As required by Dutch legislation, all animal experiments were approved in advance by the institutional Animal Welfare Committee (Erasmus University Medical Center, Rotterdam, The Netherlands). All clinical investigations were conducted according to the principles expressed in the Declaration of Helsinki. Frozen frontal cortex samples were obtained from seven C9FTD/ALS patients, two *GRN* FTD patients, one *MAPT* FTD patient, two *VCP* FTD patients and five non-*C9ORF72* non-demented control patients. Patient information regarding age of onset and disease duration can be found in Table S1.

### Constructs

Plasmids containing the pcDNA3.1+Peredox-mCherry-NLS (Addgene, 32384) or pcDNA3.1+100xGlycine-Arginine [a kind gift from the Isaacs lab (Mizielinska et al., 2013] were transformed into Top10 competent cells followed by DNA isolation using a NucleoBondXtra Maxi Kit (BioKé). Constructs were linearized by digestion with Apal (New England Biolabs) and/or NheI (New England Biolabs), purified by phenol-chloroform extraction and quantified using a nanodrop spectrophotometer (Thermo Fisher Scientific). To obtain control constructs for the injection of RNA, we performed site-directed mutagenesis to destroy the ATG start codon using a QuickChange Lightning Site-Directed mutagenesis Kit (Thermo Fisher Scientific). The following primers were used to obtain the mutated TAG stop codon: forward, 5′-CTCGTCCACG TCCCTAGGTGGGATCCG-3′; and reverse, 5′-GCTCGGATCCCACCTAGGGACGTGGAC-3′. To denature the DNA template and anneal the mutagenic primers containing the desired mutation, we used a thermal cycling protocol [1× (2 min at 95°C), 18× (20 s at 95°C, 10 s at 60°C, 3 min at 68°C), 1× (5 min at 68°C)]. After mutant strand synthesis, the template DNA was digested by adding 1 µl of the Dpn1 enzyme, and mixtures were incubated for 5 min at 37°C. Mutated DNA constructs were transformed and isolated as described above.

### RNA synthesis

For the production of RNA, an mMessage mMachine T7 Kit (Invitrogen) was used. Linear template DNA (400 µg) was mixed with 2 µl reaction buffer, 10 µl NTP/CAP, 0.1 µl RNAse out and nuclease-free water. The mixture was incubated at 37°C for 2 h. After the incubation, 1 µl TURBO DNase was added to remove template DNA at 37°C for 15 min. Lithium chloride (30 µl) and 30 µl nuclease-free water was added for precipitation, and mixtures were placed at −20°C for at least 30 min. RNA was subsequently centrifuged at 17,000 ***g*** at 4°C, washed with 70% ethanol, re-centrifuged, dried in air, dissolved in DEPC water and quantified using a nanodrop spectrophotometer. Samples were stored at −80°C in aliquots.

### Injections and fish maintenance

For all experiments, one-cell-stage zebrafish embryos of the wild type (AB) or the SecA5 YFP zebrafish reporter line (van Ham et al., 2010) were injected in the yolk sac within 30 min after egg fertilization. No fish older than 4 dpf were used and at this time zebrafish embryos are not sexually different. Injection mixtures contained a standard amount of 400 pg/nl mCherry-mRNA and 10% phenol red. The poly-GR mRNA was added to this mixture with a final concentration of 1 to 100 pg/nl. All zebrafish embryos were injected with 2 nl of injection mix. After injection, embryos were kept in a 28°C incubator for 1-4 days in E3 medium and on the first day with additional methylene blue as an antifungal aid. Propylthiouracil (1:40) was added to E3 medium to prevent pigmentation and keep the fish optically transparent. At 24 h post fertilization, the embryos underwent visual inspection for dysmorphic features. Morphologically abnormal embryos and unfertilized eggs were excluded from all further experiments. Embryos were selected at 1 dpf and deemed to be correctly injected on the basis of their mCherry signal intensity. The non-mCherry fluorescent embryos and mosaic embryos were disposed. All experiments were approved by the local animal welfare committee.

### Apoptotic cluster count

The apoptotic cluster quantification study was performed using the SecA5 YFP zebrafish reporter line (van Ham et al., 2010) at 1-4 dpf with three separate injection rounds for each timepoint, and a minimum of ten fish per group per timepoint were imaged (total *N* was 30 per group per timepoint). Prior to imaging, embryos were dechorionated, anesthetized with 10% tricaine and embedded in 1.8% low-melting-point agarose. A Leica SP5 AOBS confocal microscope and an HCX L 20× NA 1.00 water dipping objective were used for the apoptotic cluster count. A *z*-stack step size of 4.2 μm was used, ranging from the first to last YFP^+^ cell on the *Z*-axis. Automatic quantification of apoptotic cells was performed using Fiji software (https://imagej.net/software/fiji/) and the 3D object counter application with automated threshold and a minimum of three voxols. Statistical analysis was conducted using Prism (https://www.graphpad.com). One-way ANOVA with Barlett's test for equal variances showed a significant difference in the variance per group, so we performed a Kruskal–Wallis test that does not assume equal variances and a Dunn's post-hoc multiple comparison test to compare groups.

### TUNEL assay

Wild-type AB strain fish injected with 10 pg poly-GR or only 400 pg mCherry mRNA were used for TUNEL assay analysis. Fish were fixed at 2 dpf for 4 h in 4% paraformaldehyde followed by washing in PBS. Subsequently, fish were washed and kept in 100% MeOH at −20°C until further processing. Rehydration was obtained by incubating fish in decreasing concentrations (75%, 50% and 25%) of MeOH followed by PBS. After washing in PBS-T [0.2% Triton X-100 (Sigma-Aldrich) in 0.1 M PBS] fish were treated with 10 µg/ml proteinase K in PBS-T for 15 min and refixed with 4% paraformaldehyde for 20 min. After washing fish three times in PBS-T for 10 min, fish were incubated for 30 min with 100 µl TdT buffer (Click-iT Plus TUNEL Assay 647 dye, Invitrogen). Following initial incubation, fish were incubated overnight with 100 µl TdT reaction mix (94 µl TdT buffer, 2 µl EdUTP and 4 µl TdT enzyme). After incubation, fish were washed with 3% bovine serum albumin (BSA; Sigma-Aldrich) in PBS-T three times followed by incubation with 100 µl Click-iT reaction mix for 3 h and three washed with 3% BSA in PBS-T. Fish were mounted in 1.8% low-melting-point agarose (Invitrogen). Imaging was performed using a SP5 Intravital microscope equipped with an HCX-APO L 20× objective (Leica). *n* = 9 fish per group.

### Synaptic Vesicle 2 wholemount staining

At 48 h post injection with 10 pg ATG poly-GR, 10 pg TAG poly-GR, or only 400 pg mCherry mRNA, wild-type AB zebrafish were fixed overnight in 4% paraformaldehyde. Fish were permeabilized with acetone for 1 h at −20°C and blocked with 1% BSA, 1% DMSO and PBS-T for 1 h at room temperature. Subsequently, fish were stained for 30 min with 1 µg/ml α-bungarotoxin-TRITC (Invitrogen), washed with PBS and incubated overnight at 4°C with the anti-mouse IgG SV2 antibody (1:200, AB231587, Developmental Studies Hybridoma Bank, University of Iowa). After incubation, fish were washed ten times in 1.5% PBS-T and incubated overnight with the secondary antibody anti-mouse-Cy5 (1:200, Sigma-Aldrich). The following day, the fish were washed six times in 1.5% PBS-T and mounted with 1.8% low-melting-point agarose (Thermo Fisher Scientific). Imaging was performed using a Leica SP5 AOBS confocal microscope and an HCX L 20.0×1.00 water dipping objective. N was ten fish per group. The intensity of the SV2 staining was measured for *n*=5 independent fish per group, across three neurites per fish, and corrected for background fluorescence. We performed a one-tailed unpaired *t*-test with Welch's correction.

### Fluorescent immunohistochemistry

For immunohistochemical analysis, fish from the AB wild-type strain were injected with 10 pg poly-GR or only 400 pg mCherry mRNA. Fish were fixed at 1-4 dpf (*n*=30 per group) overnight in 4% paraformaldehyde and subsequently embedded in paraffin. Tissues were cut into 6-µm sections using a rotary microtome. Sections were deparaffinized using xylene and rehydrated in an alcohol series (100%, 96%, 90%, 80%, 70% and 50%). Antigen retrieval was carried out in 0.01 M sodium citrate (pH 6.0) using pressure cooker treatment. Endogenous peroxidase activity was blocked with 3% H_2_O_2_ and 1.25% sodium azide in 0.1 M PBS. Immunostaining was performed overnight at 4°C in PBS block buffer (0.1 M PBS, 0.5% protifar and 0.15% glycine) with primary antibodies (anti-GR, 1:5000, LifeTein; anti-yH2AX, 1:750, GeneTEX) at 4°C. After incubation with the primary antibody, sections were washed with PBS block buffer and incubated with secondary anti-mouse IgG/rabbit Cy2/3-linked antibodies (1:500, Jackson ImmunoResearch). To remove background staining, a 10 min incubation with Sudan Black [Sigma-Aldrich; 0.1 g in 100 ml 70% ethanol (filtered)] was performed. To visualize nuclei, slides were incubated for 10 min with Hoechst 33342 (Invitrogen). Slides were mounted with ProLongGold (Invitrogen) and kept at 4°C until imaging using a Zeiss LSM700 confocal microscope with 40× and 63× lenses.

### Protein isolation from frozen frontal cortex

Prior to lysing, frontal cortex samples were thawed on ice and supplied with RIPA buffer containing 0.05% protease inhibitors (Roche) and 0.3% 1 M dithiothreitol (DTT; Invitrogen). Samples were mechanically lysed, followed by a 30-min incubation on ice. After 30 min incubation, mechanical lysing was repeated and samples were centrifuged for 20 min at 17,000 ***g*** at 4°C, followed by three 1 min sonications. After sonication, samples were centrifuged for 20 min at 4°C and the supernatant was used for ELISA. Whole protein content was determined using a bicinchoninic acid assay (BCA; Thermo Fisher Scientific). The pellet was incubated at 95°C for 2.5 h in 150 µl 20% SDS, with 5 s vortexing at maximum intensity every 30 min. After incubation, samples were added to 500 µl 2% SDS and subjected to an ELISA.

### ELISA

Poly-GR sandwich ELISA was performed at 1-4 dpf with three separate experiments for each timepoint and 30 fish per group per timepoint. Prior to lysing, fish were dechorionated and euthanized. Mechanical lysis of fish was performed in RIPA buffer containing 0.05% protease inhibitors (Roche) and 0.3% 1 M DTT (Invitrogen). After 30 min incubation, mechanical lysis was repeated and samples were centrifuged at 13.000 ***g*** for 15 min at 4°C, followed by three 1 min sonications. After sonication, samples were centrifuged at 13.000 ***g*** for 20 min at 4°C. Whole protein content was determined using a BCA assay (Thermo Fisher Scientific). MaxiSorp 96-well F-bottom plates (Thermo Fisher Scientific) were coated for 2 h with 5.0 µg/ml monoclonal GR antibody (LifeTein Services) followed by overnight blocking with 1% BSA in PBS-Tween (0.05% Tween 20 in 0.1 M PBS) at 4°C. After washing, samples were added at 300 µg total protein in one well and 2-fold diluted in PBS in a second well. A standard curve made with 15× GR synthetic peptide was added in duplo. All samples were incubated for 1 h on the plate. After washing, all wells were incubated for 1 h with biotinylated monoclonal anti-GR antibody (LifeTein) at a final concentration of 0.25 µg/ml in PBS-Tween/1% BSA. After washing, samples were incubated for 20 min with streptavidin-horseradish peroxidase conjugate (R&D Systems) diluted 1:200 in PBS-Tween/1% BSA. Following extensive washing, samples were incubated with substrate reaction mix (R&D Systems) for 15 min and stopped using 2 N H_2_SO_4_. Readout was performed at 450 nm and 570 nm (Varioskan). We performed a two-way ANOVA with post Bonferroni test for differences between groups over time.

### Rescue experiments

Trolox (Sigma-Aldrich) was dissolved in DMSO and diluted in E3 egg medium to a final concentration of 50 μM. Dissolved Trolox or similar volumes of DMSO as a control were added to the egg water directly after micro-injections and refreshed every day. The number of apoptotic clusters was determined at 1-4 dpf as described above in SecA5 YFP zebrafish (*n*=30 per experiment from three independent experiments). One-way ANOVA with Barlett's test for equal variances showed a significant difference in the variance per group, so we performed a Kruskal–Wallis test that does not assume equal variances and Dunn's post-hoc multiple comparison test to compare groups.

### MitoSOX assay

Wild-type AB strain fish injected with 10 pg poly-GR or only 400 pg mCherry mRNA were used for MitoSOX (Invitrogen) assay analysis. As a positive control for mitochondrial stress, 2-dpf wild-type AB strain fish treated with 100 µM H_2_O_2_ were used for MitoSOX (Invitrogen) assay analyses. Fish were collected and sorted into groups of 10-15 fish. Subsequently, fish were dechorionated and washed once with Hanks's balanced salt solution (HBSS Calcium Magnesium; Gibco). Fish were incubated with 100 µM H_2_O_2_ in HBSS for 20 min at 28°C to induce stress. Following H_2_O_2_ incubation, all fish were washed three times with warmed HBSS medium (28°C). For MitoSOX staining, all fish were dechorionated and washed once with prewarmed HBSS (28°C), and incubated with 400 µl of 5 µM MitoSOX stock reagent diluted in HBSS at 28°C for 20 min. Following MitoSOX incubation, all fish were washed three times with warmed HBSS medium (28°C) and anesthetized with 1× tricaine for 5 min. Fish were mounted in 1.8% low-melting-point agarose (Invitrogen). Imaging was performed using a SP5 Intravital microscope equipped with an HCX-APO L 20×/100W objective (Leica). An *F*-test for equal variances was *P*=0.914 for mCherry versus poly-GR, so variances were not significantly different and we performed Student's *t*-test to test for differences. For poly-GR with DMSO versus poly-GR with Trolox the *F*-test was *P*=0.045, so variances were significantly different and we proceeded with Student's *t*-test with Welch's correction.

### qPCR

Transcript levels of poly-GR mRNA in injected zebrafish embryos were determined using SYBR Green fluorescence (iTaq Universal SYBR Supermix; Bio-Rad). Wild-type AB strain fish uninjected (controls) or injected with 10 pg poly-GR were used, with *n*=30 fish per group. All samples were run in triplo. Primers for the poly-GR mRNA were as follows: FW primer, 5′-CCAAGCTGGCTAGCGTTTA-3′, and RV primer, 5′-TACCTCGTCCACGTCCCAT-3′; and β-actin gene, FW primer, 5′-GCTGTTTTC CCCTCCATTGTT-3′ and RV primer, 5′-TCCCATGCCAACCATCACT-3′. Analysis was performed using the 2-ΔΔCt method with data normalized to β-actin.

## Supplementary Material

Supplementary information
