# Supplementary material for: Reduction of oxidative stress suppresses poly-GR-mediated toxicity in zebrafish embryos
Source: Dis Model Mech. 2021 Dec 1;14(11):dmm049092. doi: 10.1242/dmm.049092 (PMC8649169; doi:10.1242/dmm.049092)
Supplement: Supplementary information [file dmm-14-049092-s1.pdf]

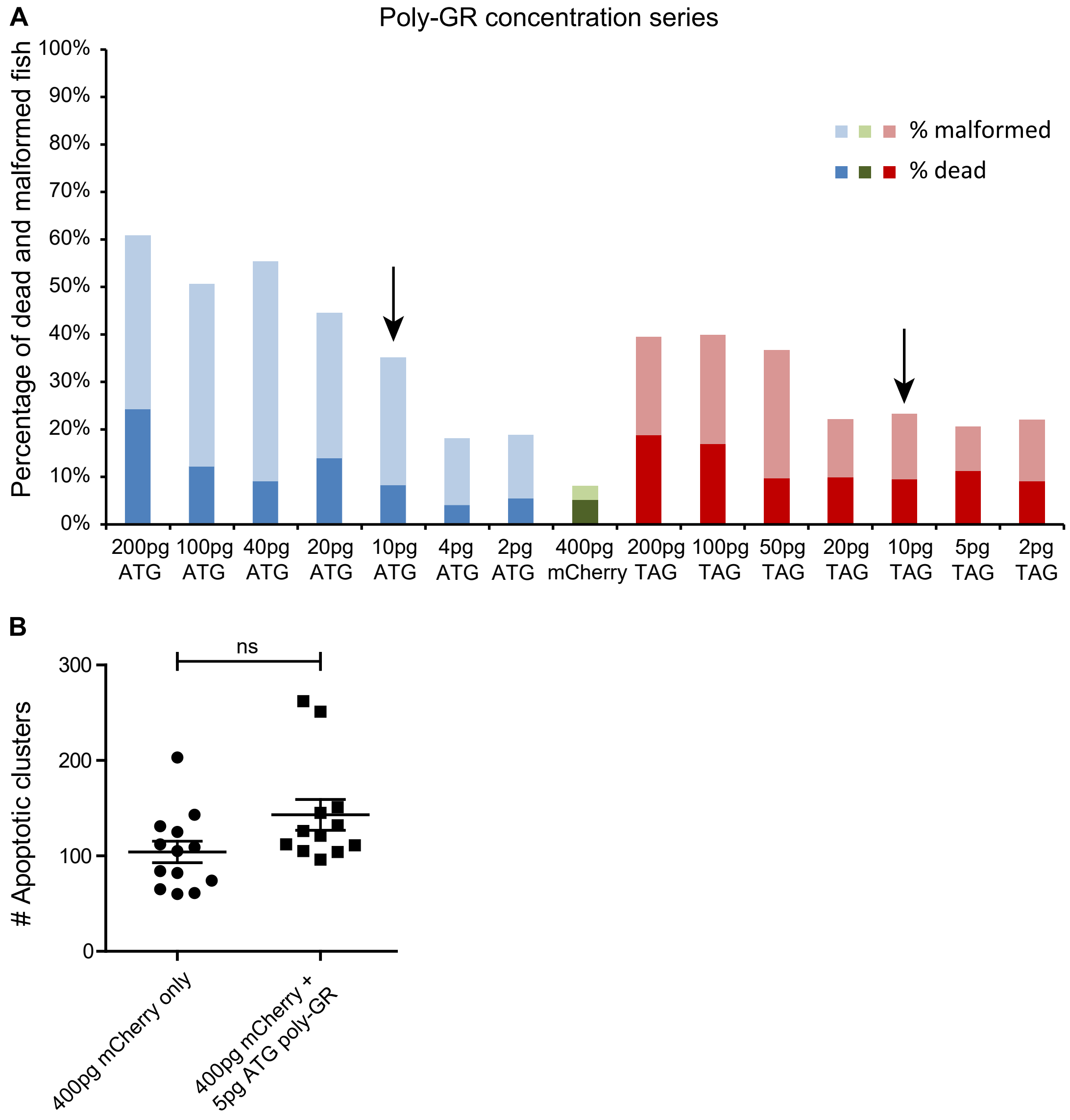

Fig. S1. Concentration series of poly-GR ATG-mediated and TAG-mediated constructs. A) Constructs were injected in the yolk sac of fertilized oocytes in concentrations ranging from 1-200pg/nl. The amount of dead and malformed fish was assessed 24 hours after injection. Malformed embryos were defined as: severely undeveloped, having no head, a smaller head or only 1 eye. Fish with heart edema and/or tail twists were also scored as malformed. The average amount in mCherry injected fish is about 5% dead and only a few % malformed fish. 10pg poly-GR injections were used for all further analysis (arrows). All malformed fish were taken out of any further analysis. N = 50 fish per construct per concentration. B) Quantification of z-stack images of Sec-A5 fluorescent reporter line embryos after injection with 5pg ATG poly-GR or 400pg mCherry only at 2 days post fertilization (dpf). Two-tailed unpaired t-test $p=0.061$ which is no significant (ns) differ-ence between mCherry only injected fish and 5pg of ATG poly-GR. Quantification of 10pg ATG poly-GR is shown in main figure 1B.

Disease Models & Mechanisms • Supplementary information

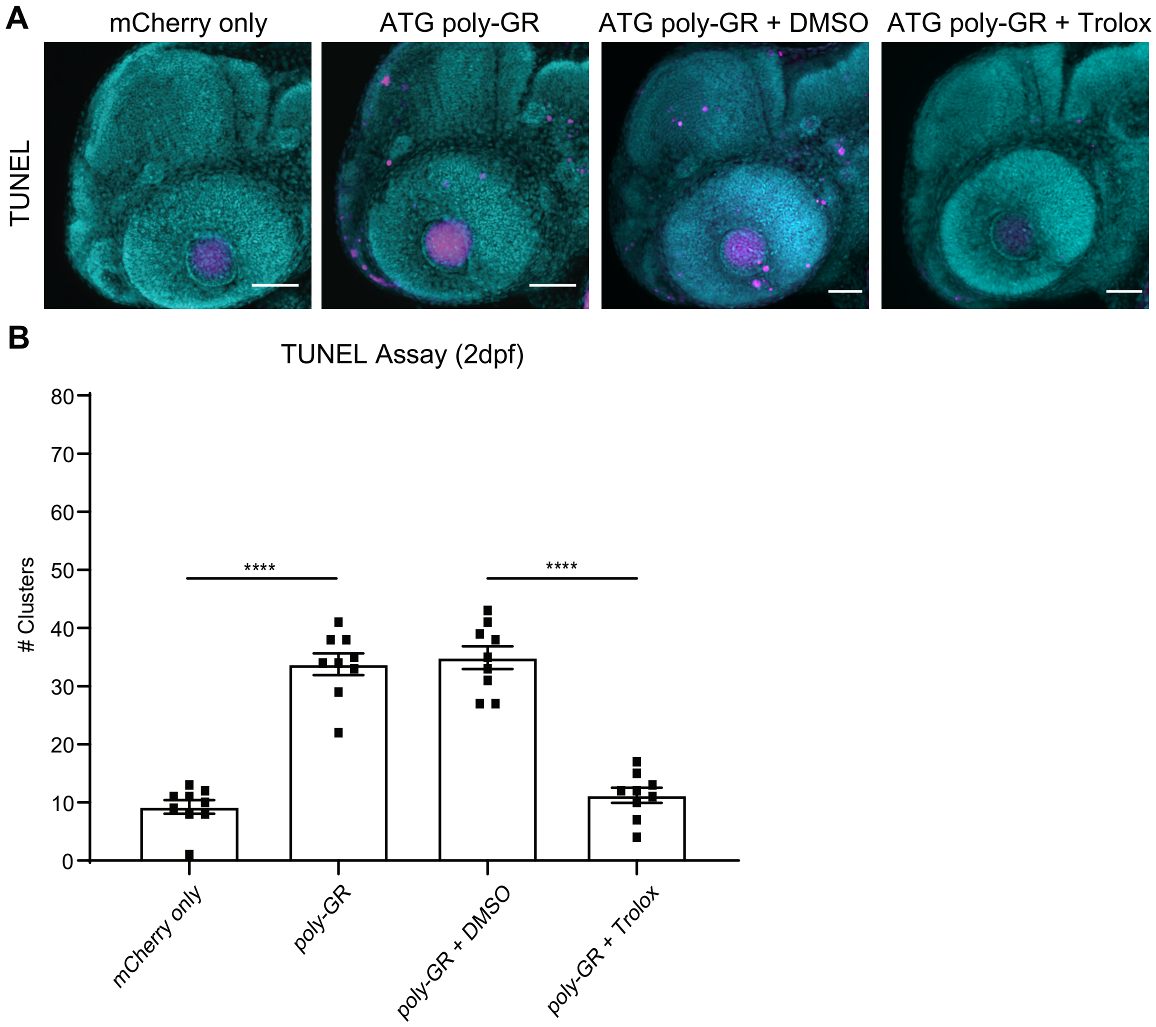

Fig. S2. Quantificaton of TUNEL assay at 2dpf. A) Max projection of z-stack images of TUNEL assay in wildtype AB embryos 48 hours after injection with 10pg poly-GR or 400pg mCherry only and treated with DMSO or Trolox (50μM, dissolved in DMSO). TUNEL positive signal is magenta, cell nuclei are stained with DAPI in blue. Scale bar is 50 μm. n = 10 per group. B) Quantification of TUNEL assay on wildtype AB embryos after injection with 10pg poly-GR or 400pg mCherry only, treated with 50μM Trolox or DMSO at 2 days post fertilization (dpf). N = 9 fish/group. 1-way ANOVA p=<0.0001. Post Tukey's test shows a significant difference between poly-GR injected fish compared to mCherry and between poly-GR injected fish treated with Trolox compared to DMSO.

Disease Models & Mechanisms • Supplementary information

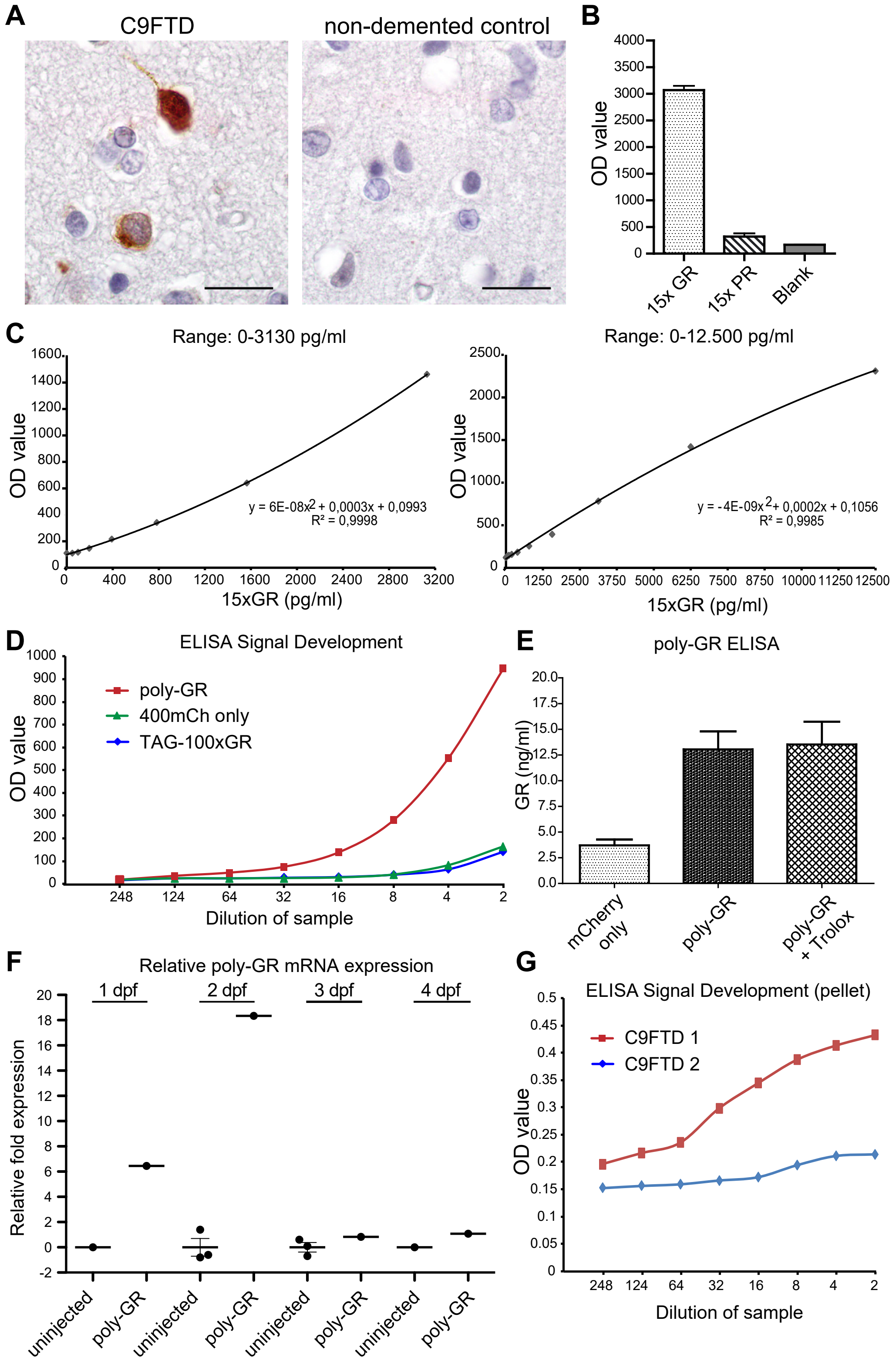

Fig. S3. Validation of ELISA for poly-GR. A) Poly-GR staining on C9FTD and non-demented control frontal cortex section shows poly-GR staining in the whole cell body or as perinuclear aggregate. Scale bars are 20μm. B) ELISA shows a positive selective signal for a synthetic 15xGR peptide over a synthetic 15xPR peptide. C) Dose-response curves for the synthetic 15xGR peptide in the low (0-3130pg) and high (1-12.500pg) range. The limit of detection in the low range is 200pg. D) ELISA signal development of a dilution series of protein samples isolated from wildtype AB embryos injected with 10pg poly-GR, 400pg of mCherry only or 10pg of the poly-GR stop construct. E) Quantification of poly-GR levels in mCherry only, poly-GR injected and poly-GR injected fish treated with Trolox. 1-way ANOVA with post Tukey test shows no significant difference between poly-GR peptide levels in Trolox treated embryos. F) Q-PCR for poly-GR mRNA levels in uninjected controls versus 10pg poly-GR injected fish. G) ELISA singal development of protein samples isolated from the pellet of the frontal cortex of 2 independent C9ORF72 FTD cases. After running the supernatant (which gave a calculated poly-GR value of 48.99 ng/ml for sample 1 and 43.39 ng/ml for sample 2), the pellet fraction was treated with anoth-er round of 20% SDS and 95°C incubation to dissolve more poly-GR, which only yield-ed 1.28 ng/ml extra for sample 1 and no extra singal for sample 2 (OD value of sample 2 remained under the blanco control OD value).

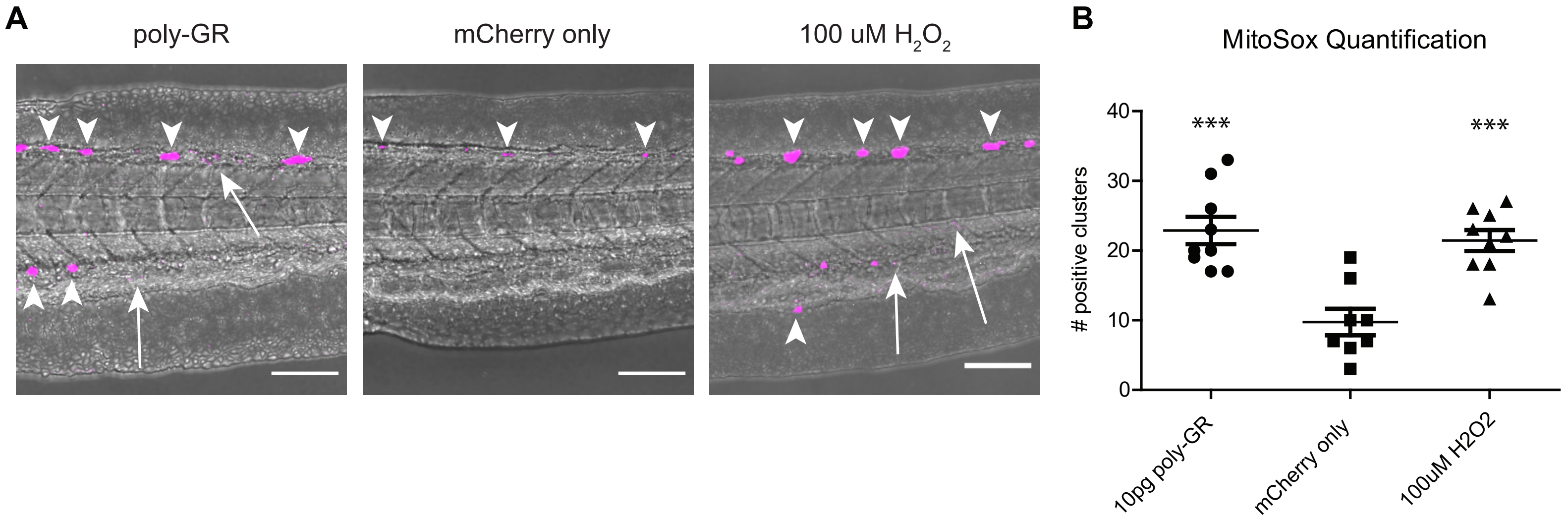

Fig. S4. MitoSOX assay in poly-GR injected embryos resembles levels of 100µM H_2O_2 treated embryos. MitoSox staining shows ROS reactivity in magenta in pigmented cells (arrowheads) but also along the tail (arrows) of H2O2 and 10pg poly-GR injected fish. Scale bar is 100µm. B) Quantification of MitoSOX fluorescence signal. *N*=9 fish per group. 1-way ANOVA p = 0.0001 with post Tukey test indicating both poly-GR injected and H_2O_2 treated embryos are significantly higher than mCherry injected embryos. poly-GR injected vs H_2O_2 treated embryos don't differ from each other.

Table S1. Patient characteristics

Patient ID	Clinical diagnosis	Genetic diagnosis	Age of onset	Disease duration in years *	Male/ Female
1	FTD	*C9ORF72*	48	10	Male
2	FTD/ALS	*C9ORF72*	60	8	Female
3	FTD	*C9ORF72*	61	7	Female
4	FTD	*C9ORF72*	71	7	Male
5	FTD	*C9ORF72*	69	6	Male
6	FTD/ALS	*C9ORF72*	39	3	Female
7	FTD/ALS	*C9ORF72*	61	2,8	Female
8	FTD	*GRN* (Gln24X)	54	5	Male
9	FTD	*GRN* (Gly387fs)	57	3	Male
10	FTD	*VCP* (p.R159S)	56	5	Female
11	FTD	*VCP* (p.T262S)	60	7	Female
12	FTD	*MAPT* (P301L)	58	8	Female
13	Non-demented	N/A	N/A	N/A	Male
14	Non-demented	N/A	N/A	N/A	Female
15	Non-demented	N/A	N/A	N/A	Male
16	Non-demented	N/A	N/A	N/A	Female
17	Non-demented	N/A	N/A	N/A	Female

* Disease duration has been estimated from the onset of first symptoms (retrospectively determined, not date of diagnosis) until death.
